# Fenarimol, a Pyrimidine-Type Fungicide, Inhibits Brassinosteroid Biosynthesis

**DOI:** 10.3390/ijms160817273

**Published:** 2015-07-29

**Authors:** Keimei Oh, Tadashi Matsumoto, Ayumi Yamagami, Tomoki Hoshi, Takeshi Nakano, Yuko Yoshizawa

**Affiliations:** 1 Department of Biotechnology, Faculty of Bioresource Sciences, Akita Prefectural University, 241-438, Shimoshinjo Nakano, Akita 010-0195, Japan; E-Mails: tmatsumoto@affrc.go.jp (T.M.); M16G027@akita-pu.ac.jp (T.H.); yyoshizawak@akita-pu.ac.jp (Y.Y.); 2 National Agricultural Research Center, National Agriculture and Food Research Organization, 3-1-1 Kannondai, Tsukuba, Ibaraki 305-8666, Japan; 3 Gene Discovery Research Group, RIKEN Center for Sustainable Resource Science, Wako, Saitama 351-0198, Japan; E-Mails: ayamagam@riken.jp (A.Y.); tnakano@riken.jp (T.N.); 4 CREST, Japan Science and Technology Agency, Kawaguchi, Saitama 332-0012, Japan

**Keywords:** brassinosteroid biosynthesis inhibitor, fenarimol, plant hormone, plant growth regulation

## Abstract

The plant steroid hormone brassinosteroids (BRs) are important signal mediators that regulate broad aspects of plant growth and development. With the discovery of brassinoazole (Brz), the first specific inhibitor of BR biosynthesis, several triazole-type BR biosynthesis inhibitors have been developed. In this article, we report that fenarimol (FM), a pyrimidine-type fungicide, exhibits potent inhibitory activity against BR biosynthesis. FM induces dwarfism and the open cotyledon phenotype of *Arabidopsis* seedlings in the dark. The IC_50_ value for FM to inhibit stem elongation of *Arabidopsis* seedlings grown in the dark was approximately 1.8 ± 0.2 μM. FM-induced dwarfism of *Arabidopsis* seedlings could be restored by brassinolide (BL) but not by gibberellin (GA). Assessment of the target site of FM in BR biosynthesis by feeding BR biosynthesis intermediates indicated that FM interferes with the side chain hydroxylation of BR biosynthesis from campestanol to teasterone. Determination of the binding affinity of FM to purified recombinant CYP90D1 indicated that FM induced a typical type II binding spectrum with a *K_d_* value of approximately 0.79 μM. Quantitative real-time PCR analysis of the expression level of the BR responsive gene in *Arabidopsis* seedlings indicated that FM induces the BR deficiency in *Arabidopsis*.

## 1. Introduction

Brassinosteroids (BRs) are plant-specific polyhydroxysteroids that are structurally similar to cholesterol-derived animal steroid hormones and the insect molting hormones ecdysteroids. The identification of BR-deficient and BR-insensitive mutants provided conclusive evidence that BRs are potent growth-promoting plant hormones. BRs play important roles in plant growth, development and responses to environmental cues [1,2]. BRs are essential regulators in cell elongation, cell division, and sex determination [3,4]. Mutants with impaired BR synthesis display dramatic growth defects, such as decreased cell elongation, resulting in pleiotropic dwarf phenotypes, delayed flowering, and male sterility [5,6,7,8]. BRs also modulate plant metabolic pathways in response to environmental biotic and abiotic stress [2,9].

As a key player involved in the control of plant architecture, the biosynthetic pathway of BRs is believed to be a good target for engineering a high yield of crops [10]. Crop plants with desirable architecture are able to produce much higher grain yields, such as in the case of the “Green Revolution” in which grain yields were significantly increased by growing lodging-resistant semi-dwarf varieties of wheat and rice [11]. In this context, reducing BR levels in plant tissues using chemicals is one feasible method to facilitate the improvement of crop yields. Furthermore, using chemicals has advantages over genetic approaches; for example, they can be applied to the plants at different stages of growth and development as well as to different plant species [12]. Moreover, agrochemicals have currently been widely used in the agricultural industry. Under this background, we have been working on the design and synthesis of inhibitors targeting BR biosynthesis [13,14,15,16].

**Figure 1:**
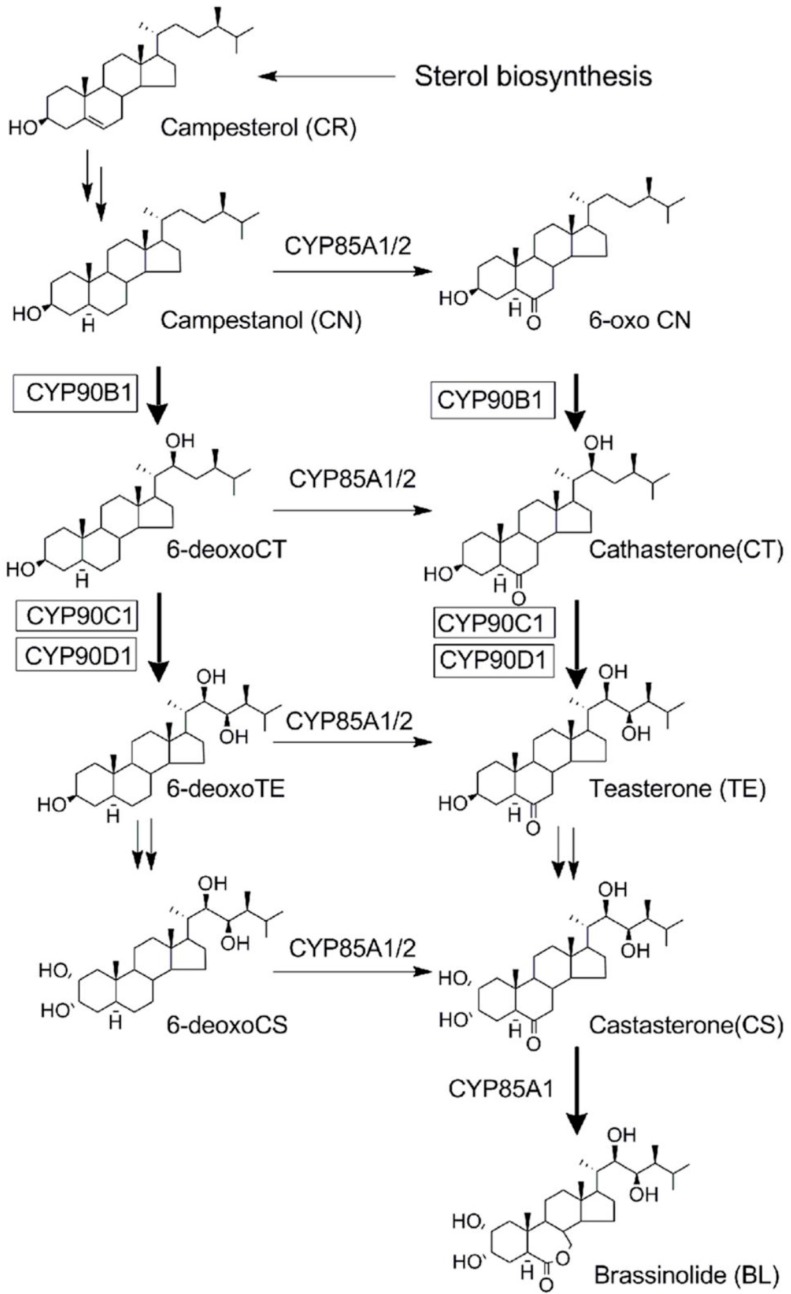
The CYP450s involved in brassinosteroids biosynthesis.

The establishment of the biosynthetic pathways (Figure 1) [17] and functional analysis of BR biosynthesis enzymes provided important information for the rational development of BR biosynthesis inhibitors. It has been demonstrated that BR biosynthesis was mediated by several cytochrome P450 monooxygenases (P450s). DWF4/CYP90B1 is thought to catalyze the C-22 hydroxylation of campesterol (CR) [18]. CPD/CYP90A1 is thought to be involved in the C-3 dehydrogenation of steroid skeletons [19]. CYP90C1/ROT3 and CYP90D1, which are genetically closely related, are shown to have redundant functions as C-23 hydroxylases [20]. *Arabidopsis* CYP85A1 and CYP85A2 were found to catalyze the C-6 oxidation reaction [21]. These observations indicate that many steps in BR biosynthesis are catalyzed by P450 enzymes. Consequently, it is reasonable to postulate that the biosynthetic pathway of BRs is an expedient target for P450 inhibitors.

The inhibition mechanisms of P450 have been studied in considerable detail [22]. The nitrogen-containing heterocyclic compounds such as pyrimidine and azole derivatives are efficient P450 inhibitors due to the intrinsic affinity of the nitrogen electron pair in heterocyclic molecules for the prosthetic heme iron. The heterocyclic compounds bind not only to the lipophilic regions of the protein but also simultaneously interact with the prosthetic heme iron [23].

**Figure 2:**
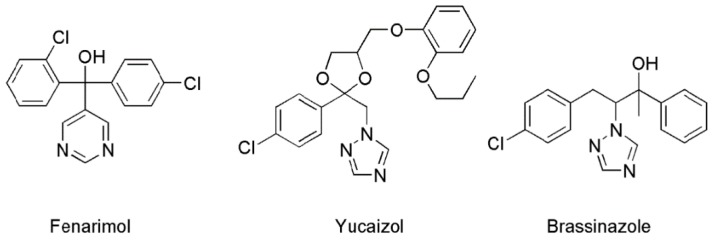
Chemical structures of BR biosynthesis inhibitors.

Based on these observations, we conducted a chemical screening of new BR biosynthesis inhibitors among the 14-α demethylase (CYP51)-inhibiting fungicides. We found that fenarimol (FM, the structure is shown in Figure 2), a fungicide used worldwide that acts as a potent inhibitor of ergosterol biosynthesis, induced phenocopy of BR-deficient mutants with short hypocotyl and open cotyledons [24]. Because the mechanisms underlying the action of FM on BR biosynthesis have not yet been elucidated, we report herein the biochemical and physiological assessments of the mechanism of action of FM on BR biosynthesis.

## 2. Results

### 2.1. Fenarimol Induces Dwarfism and De-Etiolate Phenotypes of Arabidopsis Seedlings in the Dark

**Figure 3:**
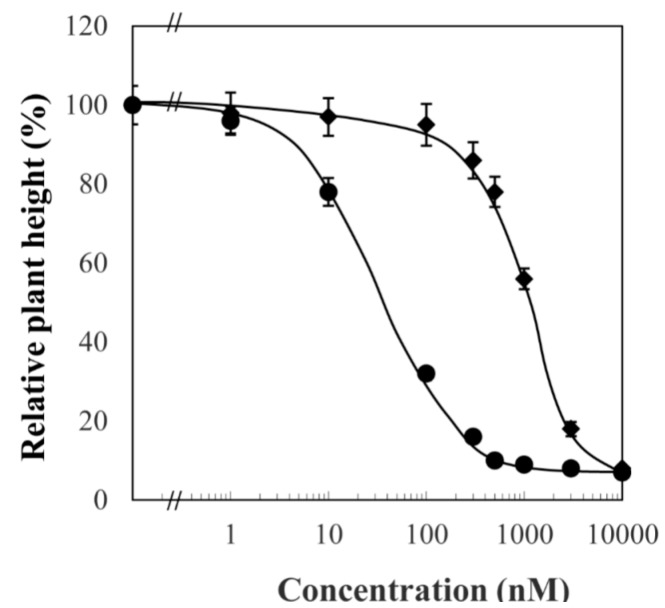
Dose-dependent effect of fenarimol and yucaizol on stem elongation of *Arabidopsis* seedlings grown in the dark. Filled cycle: yucaizol; filled diamond: fenarimol. Experiments were conducted under the conditions described in the text. Data are the means ± S.E. obtained from 8 to 10 plants. All of the experiments were duplicated to establish repeatability.

**Figure 4:**
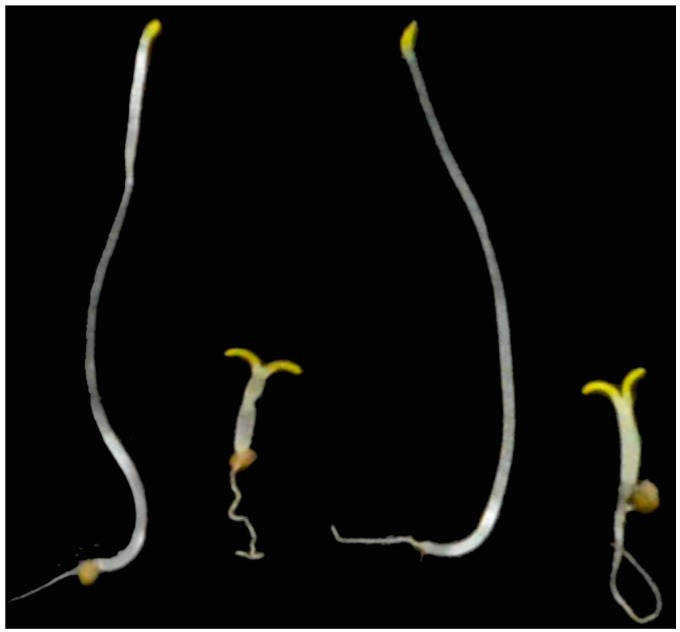
Responses of *Arabidopsis* seedlings to FM treatments and BL and GA complementation. All of the plants were grown on ½ MS media without chemical treatment in the dark for five days. Plants shown are as follows: wild-type plant without FM treatment (the plant on the left); plant grown on ½ MS media containing 10 μM FM (the second plant from the left); plant treated with 10 μM FM and 10 nM BL (second plant from the right); and wild-type plant grown on ½ MS media containing 10 μM FM and 1 μM GA (the plant on the right). Experiments were performed in triplicate to establish repeatability.

*Arabidopsis* BR-deficient mutants such as *det2* display phenotypes with short hypocotyl and open cotyledons in the dark [25]. These morphological features can be chemically induced using BR biosynthesis inhibitors [26,27]. Thus, we first tested the effects of FM on dark-grown *Arabidopsis* seedlings. In this experiment, we use yucaizol: {1-[2-(4-Chlorophenyl)-4-(2-propoxyphenoxymethyl)-[1,3]dioxolan-2-ylmethyl]-1H-[1,2,4] triazole}, a potent inhibitor of BR biosynthesis developed in our laboratory [13,14,15,16,26] (the structure is shown in Figure 2), as a positive control. Figure 3 shows the dose-response effects of FM and yucaizol on *Arabidopsis* seedlings grown in the dark. We found that the hypocotyl lengths of *Arabidopsis* seedlings treated with FM were reduced in a dose-dependent manner. The concentration of FM required for 50% inhibition on stem elongation was 1.8 ± 0.2 μM, while the IC_50_ value of yucaizol was found to be approximately 45 ± 3 nM. These results indicate that FM inhibits the hypocotyl length of *Arabidopsis* in the dark. FM also induced a de-etiolated phenotype with open cotyledons similar to BR-deficient mutants (Figure 4). These phenotypes were rescued by the application of 10 nM brassinolide but not by 1 μM GA3 (Figure 4).

### 2.2. Brz-Resistant Mutant Bzr-1-1D/bil1-1D Displays Tolerance to Fenarimol Treatment

**Figure 5:**
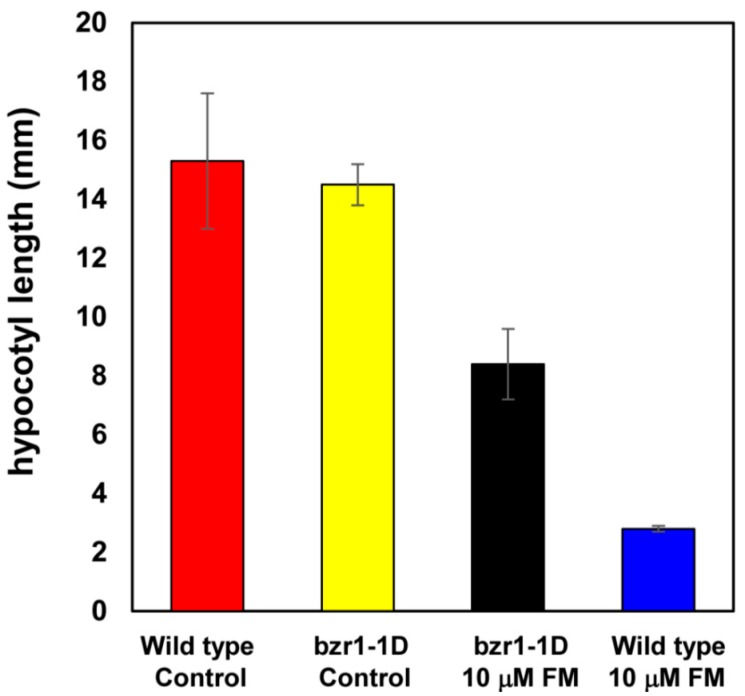
Sensitivity of *Arabidopsis* wild-type, *bzr1-1D* to FM. Seedlings of wild-type (Col-0) or *bzr1-1D* grown on ½ MS media in the dark for 5 days. Red bar: wild-type (Col-0) without FM treatment. Yellow bar: *bzr1-1D* mutant without FM treatment. Black bar: *bzr1-1D* mutant grown on ½ MS media containing 10 μM FM. Blue bar: wild-type (Col-0) grown on ½ MS media containing 10 μM FM. Data are the means ± S.E. obtained from 8 to 10 plants. All of the experiments were duplicated to establish repeatability.

Brassinazole-resistant *Arabidopsis* mutants are useful tools to determine BR biosynthesis inhibitors. Brassinazole resistant 1-1D (bzr1-1D)/Brz-insensitive-long hypocotyl 1 (bil1-1D) mutants carry a dominant gain of function mutation in transcription factor function as a major component in BR signaling. Bzr-1-1D/bil1-1D mutants exhibit a constitutive BR response even in the absence of BR [28,29]. In the present work, we then chose bzr-1-1D/bil1-1D mutants for an investigation of the properties of FM on the induction of dwarfism in *Arabidopsis* seedlings. As shown in Figure 5, in the absence of FM, the hypocotyl length of wild-type and bzr-1-1D/bil1-1D were found to be approximately 15.3 ± 2.3 and 14.5 ± 0.7 mm, respectively (the red bar and yellow bar). In the presence of FM (10 μM), the hypocotyl lengths of wild-type and bzr-1-1D/bil1-1D were found to be approximately 8.4 ± 1.2 and 3.2 ± 0.1 mm, respectively (the black bar and blue bar). These results suggest that bzr-1-1D/bil1-1D displays tolerance to FM treatment. Accordingly, data obtained in this work suggest that the inhibition of stem elongation of *Arabidopsis* seedlings by FM is due to the inhibition of BR biosynthesis.

### 2.3. Determination of the Target Site(s) of Fenarimol on BR Biosynthesis Inhibition

**Figure 6:**
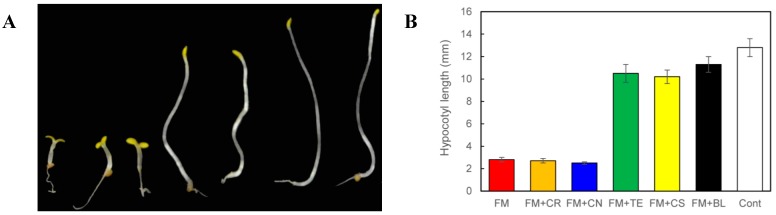
Responses of *Arabidopsis* seedlings to FM treatments and BL and BR biosynthesis intermediate complementation. Wild-type (Col-0) plants grown on ½ MS media containing designed chemicals in the dark for 5 days. The plants shown are as follows: plant grown in 10 μM FM (the plant on the left); plant treated with 10 μM FM and co-application of 100 μM CR (the second plant from the left); plant treated with 10 μM FM and co-application of 100 μM CN (the third plant from the left); plant treated with 10 μM FM and co-application of 100 μM TE (the plant in the middle); plant treated with 10 μM FM and co-application of 100 nM CS (the third plant from the right); plant treated with 10 μM FM and co-application of 10 nM BL (the second plant from the right); and the plant without chemical treatment (the plant on the right). (**B**) The hypocotyl length of corresponding plants as shown in (**A**) (*n* > 15). Data are the means ± S.E. obtained from 15 to 20 plants. All of the experiments were duplicated to establish repeatability.

To determine the target site(s) of FM on BR biosynthesis inhibition, we used a straightforward approach of feeding BR biosynthesis intermediates to FM-treated *Arabidopsis* seedlings. We thus used commercially available BR biosynthesis intermediates to determine which intermediate could rescue the FM-induced dwarfism of Arabidopsis seedlings grown in the dark. In the present work, campesterol (CR), campestanol (CN), teasterone (TE), and castasterone (CS) were selected as BR biosynthesis intermediates (Figure 6). CN was prepared by the reduction of CR by a method described previously [30]. In the presence of 10 μM FM, the hypocotyl length of *Arabidopsis* seedlings was approximately 2.8 ± 0.2 mm (Figure 6A, plant on the left, Figure 6B, red bar). When TE (100 μM, Figure 6A, fourth plant from the left, Figure 6B, green bar), CS (100 nM, Figure 6A, third plant from the right; 6B, yellow bar) and BL (10 nM, Figure 6A, second plant from the right; 6B, black bar) were applied to the growth media, the hypocotyl length of *Arabidopsis* seedlings was restored from 2.8 ± 0.2 to 10.5 ± 0.8, 10.2 ± 0.6 and 11.3 ± 0.7 mm (Figure 6A,B), respectively. This result indicates that TE, CS and BL reversed the FM-induced dwarfism of *Arabidopsis* seedlings grown in the dark, implying that the enzymes downstream of TE were not inhibited by FM (Figure 1). Application of CR (100 μM, Figure 6A, second plant from the left; Figure 6B, orange bar) or CN (100 μM, Figure 6A, third plant from the left; Figure 6B, blue bar) did not reverse the FM-induced dwarfism, as the hypocotyl length shifted from 2.8 ± 0.2 to 2.7 ± 0.3 mm and from 2.8 ± 0.2 to 2.5 ± 0.2 mm, respectively, suggesting that FM inhibits enzymes downstream of CN. Taking these results together, at least one of the target sites of FM in BR biosynthesis is the hydroxylation step from CN to TE, which is performed by CYP90B1, CYP90C1 and CYP90D1 (Figure 1) [18,20]. As in a similar experiment performed by Fujioka *et al* [25], both cathasterone and TE rescued the defective hypocotyl growth of the dark-grown *det2* mutant. Additionally, Asami *et al*. [27], used the same method to determine the target site of Brz Nevertheless, to characterize the target site of FM in the inhibition of BR biosynthesis, careful experiments should be conducted to determine the binding affinity of these enzymes to FM.

### 2.4. Fenarimol Binds to CYP90D1

**Figure 7:**
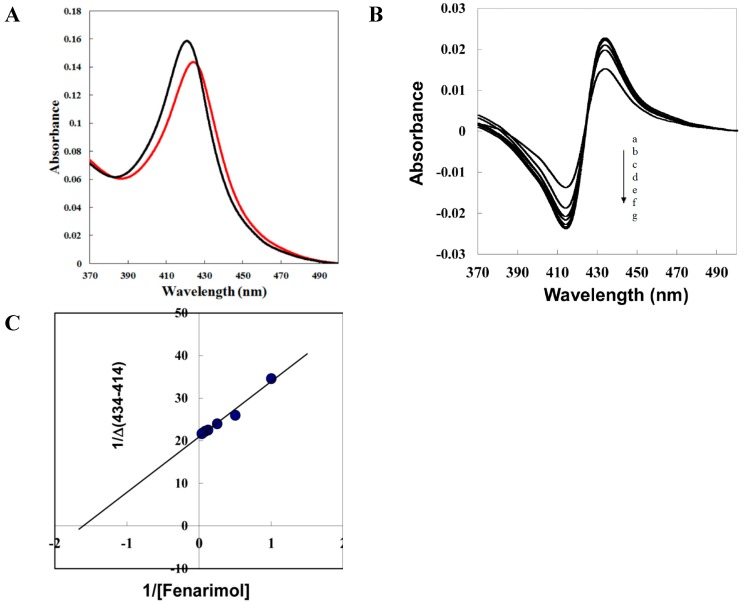
Binding of fenarimol to CYP90D1. (**A**) Absorption spectra of oxidized CYP90D1 (black line) and its fenaromol complex (red line). Recombinant CYP90D1 (3.5 μM) was dissolved in 50 mM NaH_2_PO_4_ (pH 7.0) with 0.1% Tween 20 containing 20% glycerol, and FM was added to CYP90D1 at a final concentration of 16 μM; (**B**) Spectrophotometric titration of CYP90D1 with FM induced spectral changes in CYP90D1. FM was added to CYP90D1 at various final concentrations (a, 0.7; b, 1; c, 2; d, 4; e, 8; f, 12; g, 16 μM); (**C**) The spectral dissociation constant was calculated from a double reciprocal plot of absorbance differences, ΔA (434–414 nm) *vs.* the FM concentrations given 0.79 μM. The experiment was duplicated to establish reproducibility.

To characterize the binding target responsible for FM activity in BR biosynthesis inhibition, we successfully expressed and purified CYP90D1. Thus, we determined the binding affinity of FM to CYP90D1. Binding of FM to CYP90D1 was determined by measuring optical difference spectra upon the addition of FM to recombinant CYP90D1. CYP90D1 exhibited a Soret absorption peak at 421 nm (black line Figure 7A), which is characteristic of low-spin P450s. The addition of FM to the CYP90D1 protein induced a type II absorbance shift of the heme Soret band from 421 to 426 nm (Figure 7A, black line to red line). This is characteristic of the change from a low to a high spin state of the ferric iron that is usually associated with the direct coordination of the pyrimidine group of the FM to the heme iron of CYP90D1. The dissociation constant, *K_d_*, was determined by titrating the observed spectral absorbance difference (ΔA434–A414) *vs.* the concentration of FM (Figure 7B). The double reciprocal plot for calculating *K_d_* revealed that the dissociation constant for FM was 0.79 μM (Figure 7C).

### 2.5. Molecular Function of Fenarimol for the Expression of BR-Responsive Genes

To further investigate the mechanism of action of FM in *Arabidopsis* at the molecular level, we assessed the effects of FM on the BR-responsive gene expression. Eleven-day-old wild-type plants of *Arabidopsis* grown on half MS agar-solidified media treated with or without FM were used for quantitative real-time PCR (qPCR) analysis. The concentrations of FM used were 1 and 3 μM, at which FM promisingly induces BR-deficiency-like phenotypes in *Arabidopsis* (Figure 3). We used brassinazole (the structure is shown in Figure 2, (3 μM)) as a positive control to determine the effect of FM on the regulation of BR-responsive or marker gene expression.

**Figure 8:**
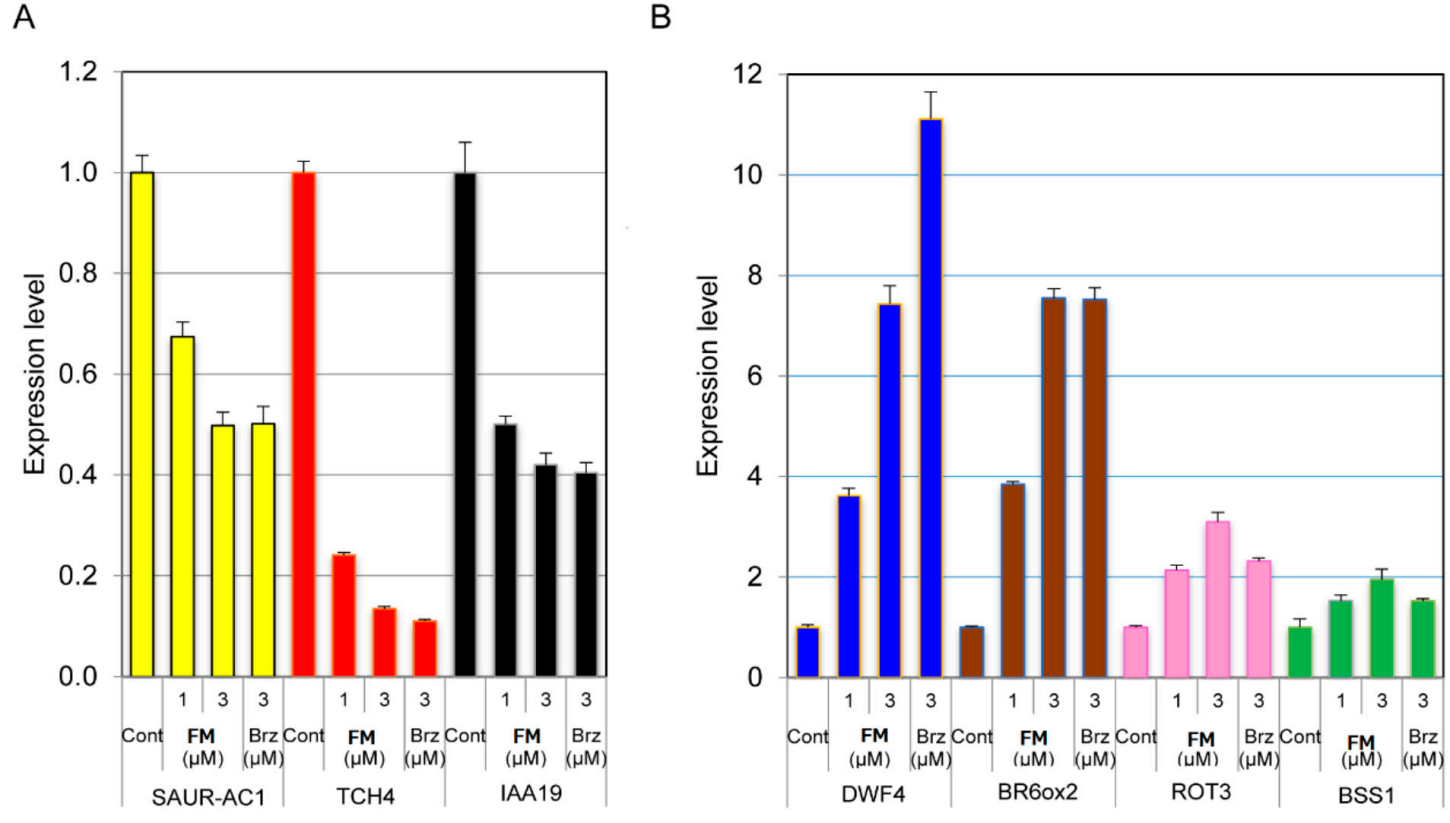

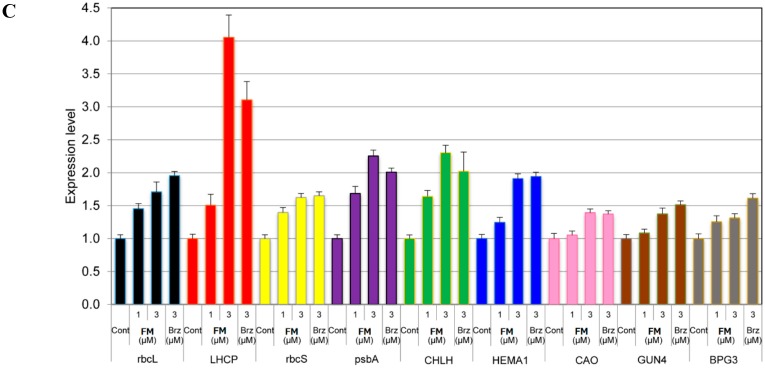
Quantitative RT-PCR experiment measuring the relative expression levels of BR-responsive genes: (**A**) Gene response to BR stimulation; (**B**) BR biosynthesis-related genes; and (**C**) photosynthesis-related genes. All results are means ± S.E., and all of the experiments were conducted three times to establish repeatability.

In the present study, we chose three marker genes whose the expression levels sensitively respond to BR stimulation: TCH4, a that gene encodes a xyloglucan endotransglycosylase, which is induced by BR treatment [31]; SAUR-AC1, an early auxin-inducible gene that is regulated by BRs independently [32]; and IAA19, which is an auxin- and BR-inducible gene [33]. We found that FM down regulated the expression level of these genes in a dose-dependent manner that displays a similar pattern to that of brassinazole (Figure 8A).

In contrast, the expression of BR biosynthesis genes (DWF4, BR6ox2 and ROT3), which are down-regulated with BR stimulation through a negative feedback mechanism [33], and BSS1, which encodes a negative regulator of BR signaling and was shown previously to be down-regulated by BRs [34], were up-regulated (Figure 8B). These results indicate that FM inhibits BR responses through a common transcriptional growth-regulatory module. The expression levels of SAUR-AC1, IAA19 and BR6ox2 in FM (3 µM) are similar to that of Brz (3 µM), which is consistent with the observation that the phenotype of FM (3 µM) is similar to that of Brz (3 µM) (Figure 8B).

BR-biosynthesis-deficient mutant *det2* and wild-type plants treated with brassinazole showed increased expression of photosynthesis genes in the dark, which is a phenomenon known as “de-etiolation in the dark” by BR deficiency [32,33,35]. In general, the photosynthesis-related genes, including rbcS, encoding the small subunit of ribulose 1,5-diphosphate carboxylase (RUBISCO) [36], LHCP, encoding light-harvesting chlorophyll a/b-binding proteins [37], rbcL, encoding the large subunit of ribulose 1,5-diphosphate carboxylase (RUBISCO) [36], psbA, encoding chlorophyll binding protein D1, and key genes involved in chlorophyll biosynthesis (CHLH, HEMA1, CAO, GUN4 and BPG3) [38] are used as genetic markers for light and BR-deficiency responses [27]. Eleven-day-old wild-type Arabidopsis plants grown on half MS agar-solidified media treated with or without FM were used for qPCR analysis. As shown in Figure 8C, under dark conditions, the expression levels of these photosynthesis genes were higher in wild-type Arabidopsis treated with FM than those without FM treatment. In addition, the positive control of Brz-treated wild-type Arabidopsis displayed similar patterns in the up- and down-regulation of the expression of these genes (Figure 8C).

## 3. Discussion

In the present work, we used a variety of methods to investigate the mechanism of action of FM and presented evidence that FM is a potent and specific inhibitor of BR biosynthesis. FM induces dwarfism of Arabidopsis seedlings grown in the dark with an open cotyledon phenotype. This morphological characteristic is similar to BR-deficient mutants of Arabidopsis [25,26,35]. To distinguish the primary site of action of FM between BR biosynthesis and GA biosynthesis, we applied brassinolide and GA to FM-treated Arabidopsis. We found that brassinolide rescued the FM-induced dwarf phenotype of Arabidopsis seedlings, while GA did not (Figure 4). Furthermore, through use of the bzr-1-1D/bil1-1D mutant, we found that bzr-1-1D/bil1-1D displays tolerance to FM treatment. This result indicated that the dwarfism of Arabidopsis seedlings induced by FM is due to the inhibition of BR biosynthesis. To identify the target of FM in BR biosynthesis, we conducted a feeding experiment involving the application of BR biosynthesis intermediates to FM-treated Arabidopsis. Data obtained in the present work demonstrated that FM targets the side chain hydroxylation located between campestanol and teasterone (Figure 5). Analysis of the binding affinity of FM to CYP90D1, which is responsible for the C23 hydroxylation of BR, indicated that FM induced a typical type II binding spectrum against CYP90D1 with a dissociation constant of approximately 0.79 μM (Figure 7C). This result provided conclusive evidence that FM targeted the step(s) of the side chain hydroxylation of BR biosynthesis.

CYP90C1 and CYP90D1 have been demonstrated to be two closely related genes with redundant functions as C-23 hydroxylases in BR biosynthesis [20]. The disruption of CYP90C1 in the *rot3* mutants results in a weak dwarf phenotype [39] but causes no appreciable alteration of the endogenous level of BL [40]. Likewise, CYP90D1 deficiency does not show any visible morphology changes in Arabidopsis [40]. However, the double mutant of CYP90C1 and CYP90D1 exhibits a severe dwarf phenotype [20,40]. As shown in the present study (Figure 4), FM induced a remarkable dwarf phenotype in Arabidopsis, and we anticipate that FM blocks C-23 hydroxylation of BR biosynthesis, thereby interfering with both CYP90C1 and CYP90D1 (Figure 1). Another possibility is that FM may inhibit CYP90B1, which is involved in the C22 hydroxylation of BRs. Because the soluble recombinant proteins of CYP90B1 and CYP90C1 were poorly expressed in our expression system, the binding analysis for FM and CYP90B1 and CYP90C1 could not be performed in the present study. However, based on the observations of the FM-induced morphological changes in Arabidopsis seedlings, combined with the observations from the feeding experiment of BR biosynthesis intermediates, we concluded that FM inhibits BR biosynthesis through interfering with the side chain hydroxylation of BRs.

Another line of evidence indicating that FM is a potent and specific inhibitor of BR biosynthesis is obtained from the qPCR analysis of BR marker gene expression. The expression levels of TCH4, SAUR-AC1 and IAA19, which are induced by BR treatment, were down-regulated by FM treatment. Furthermore, the expression of DWF4, BR6ox2 and ROT3, which are genes sensitively suppressed by BR treatment, were up-regulated by FM treatment (Figure 7A,B). FM also induced promotion of greening in the light-grown plants (Supplementary Figure S1). It has been reported that the BR-biosynthesis-deficient mutant *det2* and brassinazole-treated Arabidopsis wild-type plants induced expression of photosynthesis genes both at the germination stage in the dark and at the promotion of leaf greening in the light-grown stage [25,26,35]. The deficiency of BRs has been considered to be a unique phenomenon that could cause de-etiolation and the induction of photosynthesis gene expression in the dark [26,35]. Considering these observations, data obtained in the present work indicate that the induction of photosynthesis genes (Figure 8C) in the dark by FM was largely or exclusively due to the primary action of FM on inhibiting BR biosynthesis.

Brassinazole has been identified as the first synthetic small-molecule compound targeting C-22 hydroxylase (DWF4) in BR biosynthesis (Figure 1) [41]. Yucaizol, which was developed in our laboratory, has been recognized as a new BR biosynthesis inhibitor [26]. Data obtained in this work provide evidence for the first time that FM targets the side chain hydroxylation of BRs in BR biosynthesis. Additionally, one of the most important findings in this work is our identification of a pyrimidine-type BR biosynthesis inhibitor. The structure-activity relationships of triazole-type BR biosynthesis inhibitors, such as brassinazole and yucaizol, have been studied in considerable detail. The structural requirements of pyrimidine compounds for BR biosynthesis inhibition remain to be investigated. We expect that further structure-activity relationship studies may lead to discovery of a new class of BR biosynthesis inhibitors with a pyrimidine scaffold.

Small molecules targeting the biosynthesis of BRs are useful tools in discerning the functions of BRs [27]. They can be applied in genetic screens of mutants that involve the biological functions of BRs, and this method has emerged as a useful strategy to study biological systems in plants [28]. In the last century, mutagenesis strategies have played a central role in elucidating biological processes. These strategies have been involved in investigating the relationships between genes and phenotypes. However, the problems associated with mutagenesis strategies include genetic lethality, redundancy and tissue/development-specific expression. Instead of targeting genes, small molecules target proteins by modulating their functions; hence, the use of small molecules can overcome the problems associated with mutagenesis strategies. Another advantage of small molecules over mutagenesis strategies is that they are easy to apply to different plant species and at different stages of plant growth and development. Data obtained from the present work indicated that FM exhibits potent biological activity upon inducing BR-deficient-like phenotypes in Arabidopsis. In addition, different from triazole-type BR biosynthesis inhibitors, FM is a pyridine-type BR biosynthesis inhibitor. Therefore, further experiments using FM to explore the biological processes related to BRs may provide new insights into the detailed mechanism of BR biosynthesis and its functions.

## 4. Experimental Section

### 4.1. Chemicals

Fenarimol, GA_3_ and campesterol were purchased from Wako Pure Chemical Industries, Ltd. (Tokyo, Japan). Brassinolide, castasterone, and teasterone were purchased from Burashino Co., Ltd. (Toyama, Japan). Campestanol was prepared by reduction of campesterol using a method as described previously [30]. All of the chemicals used for biological studies, unless otherwise described, were dissolved in DMSO and stored at −30 °C before use.

### 4.2. Plant Growth Conditions and BR Biosynthesis Inhibition Assay

Seeds of *Arabidopsis* (Columbian ecotype) were purchased from Lehle Seeds (Round Rock, TX, USA). The seeds used for assay were sterilized in 1% NaOCl for 20 min and washed with sterile distilled water. Seeds were sown on a 1% solidified agar medium containing half Murashige and Skoog salt in agripots (Kirin Brewery. Co., Tokyo, Japan), with or without chemicals. Plants were grown under 16 h light (240 μE/m^2^s) and 8 h dark conditions in a growth chamber. For the dark condition, agripots were wrapped in four layers of aluminum foil. The biological activities of the test compounds were measured 5 days after sowing the seeds. Stock solutions of all of the chemicals were dissolved in DMSO to a designated concentration and applied to growth media at 0.1% (*v*/*v*).

### 4.3. Quantitative Real-Time PCR

The methods for total RNA isolation, cDNA synthesis, and RT-PCR have been previously described [42]. ACT2 and UBQ2 were used as an internal control in the light and the dark, respectively. Gene-specific primers are listed in Supplementary Table S1.

### 4.4. Construction of CYP90D1 Expression Vectors

*Arabidopsis* full-length cDNA was provided by the RIKEN BRC through the National Bio-Resource Project of the MEXT (Ministry of Education, Culture, Sports, Science, and Technology), Japan [43,44]. The expression vector pCold-GST was obtained from Kojima, C. of Osaka University [45]. The DNA fragment encoding CYP90D1 mature protein was generated by PCR with forward primer 5′-aatcgagctcatggacacttcttcttcacttttg-3′ and reverse primer 5′-ttgactgcagttatattcttttgatccaaatgggt-3′. The PCR product was digested with *Sac*I-*Pst*I and was inserted into the pCold-GST expression vector. All of the constructed plasmids were transferred to the BL21 star (DE3) strain of *E. coli* (Invitrogen, Carlsbad, CA, USA). The transformed cell was incubated in 10 mL of Luria broth containing 100 μL/mL of chloramphenicol overnight at 37 °C. Then, 10 mL of pre-culture was incubated in 1000 mL of Luria broth containing 100 μL/mL of ampicillin at 37 °C.

### 4.5. Expression and Purification of Recombinant CYP90D1

The expression and purification of recombinant CYP90D1 were performed as described previously [46]. The purified CYP90D1 was dialyzed for 6 h at 4 °C using an oscillatory dialysis system (Daiichi Pure Chemicals, Co., Ltd., Tokyo, Japan) against a 2 × 300 mL dialysis buffer (50 mM sodium phosphate buffer, pH 7.0). Cleavage of the fusion protein was carried out using HRV3C protease according to the supplier’s protocol (Takara, Bio. Co., Ltd., Shiga, Japan). Protein measurements were performed using a Protein Assay Kit (Bio-Rad, Hercules, CA, USA), using bovine serum albumin as the standard. The relative purity of recombinant CYP90D1 was estimated by SDS-polyacrylamide gel electrophoresis (12% polyacrylamide), and staining of gels was performed with Coomassie Brilliant Blue R250.

### 4.6. Binding Assay of Fenarimol to Recombinant CYP90D1

Binding of FM to CYP90D1 was measured by optical difference spectroscopy of purified recombinant CYP90D1 using a Shimadzu UV3100 spectrophotometer as we previously described [2]. Data obtained were used to calculate binding constants based on a linear regression analysis. Spectral determinations were performed at least twice for each experiment, confirming the reproducibility with respect to the spectral profile and the position of λ max and λ min.

## Supplementary Materials

Supplementary materials can be found at http://www.mdpi.com/1422-0067/16/08/17273/s1.
