# Supplementary material for: Fenarimol, a Pyrimidine-Type Fungicide, Inhibits Brassinosteroid Biosynthesis
Source: Int J Mol Sci. 2015 Jul 29;16(8):17273–88. doi: 10.3390/ijms160817273 (PMC4581192; doi:10.3390/ijms160817273)
Supplement: Supplementary file 1 [file ijms-16-17273-s001.pdf]

Supplementary Information

Table S1. Primers used in quantitative RT-PCR.

Genes	Forward Primer	Reverse Primer
ACT2 (AT3G18780)	CGCCATCCAAGCTGTTCTC	TCACGTCCAGCAAGGTCAAG
UBQ2 (AT2G36170)	CCAAGATCCAGGACAAAGAAGGA	TGGAGACGAGCATAACACTTGC
SAUR-AC1 (AT4G38850)	GAGATATGTGGTGCCGGTTT	GTATTGTTAAGCCGCCCATT
TCH4 (AT5G57560)	CGAGTCTTTGGAACGCTGAT	CTTCTTGTTGAAAGCCACGG
IAA19 (AT3G15540)	GAAGGACTCGGGCTTGAGAT	GACGCCGCTTTCACATTG
BSS1 (AT3G57130)	CATGACCTAACCTCGACTTT	CCCATCACCATTAGCTTC
DWF4 (AT3G50660)	CATAAAGCTCTCTTCAGTCACGA	CGTCTGTTCTTTGTTTCCTAA
BR6ox2 (AT3G30180)	GGAGGTGGAGTTAGGCTTTGCCC	TCTTCTCCATTCTCTTCCCATCTAT
ROT3 (AT4G36380)	GGTGGTGGGCAAAGGCTAT	GTCTTCCTCAGCCGTCCAAC
rbcL (ATCG00490)	TACTGGTACATGGACAACTG	GGTCTAAGGGATAAGCTACA
LHCP (AT1G50900)	ATCCGACCGAGTCAAGTACT	GGTTCCTTGCGAATGTCT
rbcS (AT1G67090)	GCACCGACTCCGCTCA	TGGACTTGACGGGTGTTGTC
psbA (ATCG00020)	CTATACAACGGCGGTCCTC	GCATACCCAGACGGAAACTA
CHLH (AT5G13630)	TGGTAGAGAGACAGAAGCTCGAAA	CCAAAGAACCTGCCCAAGAG
HEMA1 (AT1G58290)	GTGAGCTCTCTGCTTCTTCTGATTCTG	CTGCTTCTTTCCTTTGTATATCGATCAGCT
CAO (AT1G44446)	CCGGTGGAACACGGTTTACTTCTAGATA	AGTATCCTTGGAGACCCGAGGTAGGTGT
GUN4 (AT3G59400)	GAAACCGCGACCATATTCGAC	CGGCTTCTCCGGATATCTGAA
BPG3 (AT2G40400)	TCCAAATCCAAACGTCGTCA	CGGTGGTGCGACGAGAAT

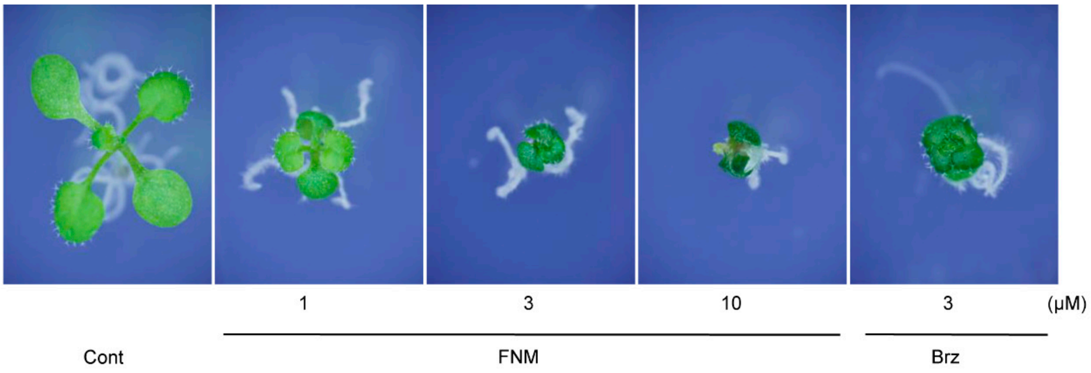

Figure S1. Responses of Arabidopsis seedlings to FM treatments and brassinazole (brz). Wild-type (Col-0) plants grown on ½ MS media containing designed chemicals in the light for 12 days. The plants shown are as follows: The plant without chemical treatment (the left plant); plant grown in 1 μM FM (the second left plant); plant treated with 3 μM FM (the third left plant); 10 μM FM (the second right plant) and plant treated with 3 μM brz (the third left plant). All of the experiments were duplicated to establish repeatability.
